# Expression of pRb and p16INK4 in human thymic epithelial tumors in relation to the presence of human polyomavirus 7

**DOI:** 10.1186/s13000-015-0418-6

**Published:** 2015-11-04

**Authors:** Marlies Keijzers, Dorit Rensspiess, Sreedhar Pujari, Myrurgia A. Abdul-Hamid, Monique Hochstenbag, Anne-Marie Dingemans, Anna Kordelia Kurz, Anke Haugg, Jos. G. Maessen, Marc H. De Baets, Axel zur Hausen

**Affiliations:** Department of Cardiothoracic Surgery, Maastricht University Medical Centre, Maastricht, The Netherlands; Department of Pulmonology, Maastricht University Medical Centre, Maastricht, The Netherlands; Department of Pathology, Maastricht University Medical Centre, P. Debyelaan 25, P.O. Box 5800, 6202 AZ Maastricht, The Netherlands; GROW-School for Oncology and Developmental Biology, Maastricht University Medical Centre, Maastricht, The Netherlands; Department of Internal Medicine IV, University Hospital Aachen, Aachen, Germany; Department of Neuro-Science, Maastricht University, School of Mental Health and Neuroscience (MHeNS), Maastricht, The Netherlands

**Keywords:** Thymic epithelial tumors, Human polyomavirus 7, pRB, p16, Viral tumorigenesis

## Abstract

**Background:**

We have recently reported the presence of the Human polyomavirus 7 (HPyV7) in human thymic epithelial tumors as assessed by diverse molecular techniques. Here we report on the co-expression of p16, retinoblastoma protein (pRb) and phosphorylated retinoblastoma protein (phospho-Rb) in human thymic epithelial tumors in relation to HPyV7.

**Methods:**

PRB, phospho-RB and p16 expression was assessed by immuno-histochemistry in 37 thymomas and 2 thymic carcinomas. 17 thymomas (46 %) and 1 thymic carcinoma (50 %) were recently tested positive for HPyV7. In addition, 20 follicular hyperplasias were tested.

**Results:**

Expression of pRb was observed in 35 thymomas (94.6 %), in 16 thymomas (43.2 %) the expression was strong. Phospho-Rb was observed in 31 thymomas (83.8 %). 19 thymomas (51.4 %) showed immunoreactivity for p16 of which 8 thymomas revealed very strong p16 expression. No p16 expression was detected in thymic carcinomas. In addition, no significant correlation between the presence of HPyV7 and pRb-, phospho-Rb- and p16-expression could be established. No correlation between pRb, phospho-Rb, p16 and WHO staging, Masaoka-Koga staging or the presence of MG was found. All 20 follicular hyperplasias showed expression of pRb and less expression of phospho-Rb.

**Conclusions:**

Although polyomaviruses have been shown to interact with cell cycle proteins no correlation between the presence of HPyV7 and the expression of pRb, phospho-Rb and p16 in human thymic epithelial tumors was observed. In as much HPyV7 contributes to human thymomagenesis remains to be established. Our data indicate pRb, phospho-Rb and p16 expression are rather unlikely to be involved in HPyV7 related thymomagenesis.

## Background

Thymomas are rare tumors arising from thymic epithelial cells. Frequently there is an association with autoimmune diseases, most often (24.5–40 %) with Myasthenia Gravis (MG) [1]. The aetiology of thymomas is unknown though many studies focus on the role of viruses testing diverse histological subtypes of thymic epithelial tumors [1–3]. In mouse strains C3H/BiDa and AKR the polyomavirus strain PTA induces thymomas, [4, 5]. We have recently reported the presence of the Human Polyomavirus 7 (HPyV7) in a large number of human thymic epithelial tumors by fluorescence in situ hybridization (FISH), immunohistochemistry (IHC) and polymerase chain reaction (PCR). [6]. The human polyomavirus 7 (HPyV7) was recently detected in 2010 from skin samples and prior to our report no other human disease had been associated with the presence of HPyV7 [7].

The human polyomavirus family is currently growing very fast [8–10], however, only the Merkel cell polyomavirus (MCPyV) has yet been identified as a novel human tumor virus in Merkel cell carcinomas (MCC), which is a very aggressive skin cancer of elderly and immune suppressed patients [11]. MCPyV is found in more than 80 % of MCC’s and its DNA is clonally integrated in the tumor genomes [11, 12]. In addition, MCPyV harbours tumor specific mutations of the large T antigen (LTag) [13]. MCPyV is supposed to induces tumorgenesis via truncated large T antigen (LTag) and small T antigen (STag) possibly inhibiting the tumor suppressor protein retinoblastoma (pRb) and protein 53 (p53) [14–16]. It has been demonstrated that the polyomavirus simian virus (SV 40) interacts through large T antigen in the cell cycle by the binding of pRb and p53 [17]. Recently it has been proposed that the LTag from WU polyomavirus, human polyomavirus 6, HPyV7 and Malawi polyomavirus might interact with p53 and pRb [18, 19]. Human papilloma virus (HPV), another potent small DNA tumorvirus is one of the most important viral causes of human cancer, and shares with MCPyV a homolog LxCXE motif in the encoded RB binding site [13, 20]. Although HPV could not be related to thymomagenesis increased transcript expression of p16 (cyclin-dependent kinase inhibitor 2A) was reported in human thymomas [21]. P16 is frequently used as a surrogate marker for HPV infection in HPV related cervical and oropharyngeal cancers [22]. Of interest, only very limited data are available regarding the possible role of pRB and p16 in human thymomas [23, 24]. In the present study we aimed to analyse the expression of pRB and p16 in human thymic epithelial tumors in relation to the presence of HPyV7.

## Methods

### Patients and tissue

Formalin-fixed and paraffin-embedded (FFPE) resection specimens were included as previously described [6]. In total 37 thymomas and 2 thymic carcinomas (19 females and 18 males; mean age 58.3 years; range 34–82 years), 20 follicular hyperplasias (15 females, 5 males, and mean age 27.4 years) of which 19 were diagnosed with MG, were included in this study. Thymomas were classified according to the world health organization (WHO) classification in thymoma type A, type AB, type B1, B2, B3 or thymic carcinoma [25]. The Masaoka-Koga classification was used to define the invasiveness of a thymoma [26]. Clinico-pathological data of thymoma and thymic carcinoma patients are summarized in Tables 1 and 2. All specimens were obtained from the Maastricht Pathology Tissue Collection (MPTC). All use of tissues and patient data was in agreement with the Dutch Code of Conduct for Observational Research with Personal Data (2004) and Tissue (2001, www.fmwv.nl).

**Table 1:** Summary of clinico–pathological data and results of thymomas

Clinicopathological data								HPyV7	P16	ProteinRb	
Lab ID	G.	Age	MG	Thym. type	Masaoka–Koga	Anti–AChR	IS/Ster.	IHC 2 t10		Anti–Rb	phosRb
1–1	F	73	+	B1/B2	I	+	+	–	–	+	+
1–2	F	75	+	B1/B2	I	+	+	–	–	+	+
1–11	F	34	+	B2	I	+	–	–	++	+	+
1–12	F	36	+	A	I	–	+	–	–	–	–
1–16	F	34	+	A	I	+	+	–	–	+	+
1–17	M	69	+	AB	I	+	+	–	(+)	+	+
1–18	F	47	+	AB	I	+	+	–	(+)	(+)	(+)
1–19	M	68	–	AB	I	NA	NA	+	+	(+)	(+)
1–21	F	38	+	AB	I	+	–	–	–	+	–
1–22	M	65	+	AB/B2	I	+	–	–	++	(+)	(+)
1–28	M	38	–	B1	I	NA	NA	–	(+)	+	+
1–31	F	82	+	A	I	+	+	+	–	(+)	–
1–32	M	47	+	B2	I	+	–	–	(+)	+	+
1–34	M	59	–	AB	I	NA	NA	+	+	+	+
1–36	F	82	–	AB	I	NA	NA	+	+	(+)	(+)
1–39	F	78	–	B1	I	NA	NA	–	+	+	+
1–43	F	78	–	AB	I	NA	NA	(+)	–	+	+
1–23	M	37	+	AB	IIA	+	–	+	(+)	+	+
1–24	M	68	+	B2	IIA	+	–	+	(+)	+	+
1–33	F	43	+	B2	IIA	+	–	+	–	(+)	+
1–35	M	45	+	AB	IIA	+	–	+	–	+	(+)
1–37	M	77	+	AB	IIA	+	–	(+)	(+)	+	+
1–38	F	80	+	AB	IIA	+	+	–	–	–	+
1–3	F	57	+	B2/B3	IIB	+	–	+	–	(+)	(+)
1–5	M	37	+	B3	IIB	+	+	+	–	(+)	(+)
1–7	F	79	+	A	IIB	–	–	(+)	–	(+)	(+)
1–8	M	58	+	A/B2	IIB	+	–	(+)	(+)	(+)	–
1–9	M	64	–	B2	IIB	NA	NA	(+)	–	+	(+)
1–15	F	53	+	B3	IIB	+	+	–	++	+	+
1–26	M	65	–	AB	IIB	NA	NA	–	+	+	–
1–42	F	57	+	AB	IIB	+	–	–	+	+	+
1–10	F	73	+	B3	III	+	+	–	–	+	+
1–13	F	54	–	B2	III	NA	NA	(+)	+	+	+
1–20	M	64	–	B3	III	NA	NA	–	–	+	–
1–41	M	65	–	B3	III	NA	NA	+	(+)	+	+
1–4	M	40	+	B2	IVA	+	+	(+)	–	+	(+)
1–30	M	37	+	B2	IVA	+	+	–	–	+	+
								17/37	19/37	35/37	31/37
								46 %	51.4 %	94.6 %	83.8 %

*Lab ID* laboratory identification, *G* gender, *MG* myasthenia gravis, *Thym. type* thymoma type, *anti–AChR* anti–acetylcholine receptor antibodies, *IS/Ster* immunosuppression/steroids, *HPyV7* human polyomavirus 7, *IHC* immunohistochemistry using 2 t10 monoclonal antibody directed against LTag of HPyV7, − negative, (+) weak positive, + positive, ++ strong positive expression, +++ very strong positive expression, *NA* not applicable

P16 expression – = < 1 %; (+) = 1 %; + = 1–25 %; ++ = > 25 %

**Table 2:** Summary of clinico–pathological data and results of thymic carcinomas

Clinicopathological data								HPyV7	P16	ProteinRb	
Lab ID	G.	Age	MG	Thym. type	Masaoka–Koga	Anti–AChR	IS/Ster.	IHC 2 t10		Anti–Rb	phosRb
1–6	M	60	–	C	III	N/A	N/A	(+)	–	+	+
1–26	F	67	–	C	IVB	N/A	N/A	–	–	+	+

*C* thymic carcinoma, *G* gender, *MG* myasthenia gravis, *anti–AChR* anti–acetylcholine receptor antibodies, *IS/Ster* immunosuppression/steroids, *NA* not applicable, *HPyV7* human polyomavirus 7, *IHC* immunohistochemistry using 2 t10 monoclonal antibody directed against LTag of HPyV7, − negative, (+) weak positive, + positive, ++ strong positive expression

### Immunohistochemistry

PRb and phosphoralized Rb (phospho-Rb) expression was detected by using two monoclonal Retinoblastoma antibodies: pRb (a.a. 332–344), clone G3-245, Pharmingen, dilution 1:300 and phospho-Rb, clone D20B12, dilution 1:100. P16 expression was performed with a monoclonal antibody (clone JC8, dilution 1:400) (Santa Cruz). Secondary antibody detection and visualization were done with the EnVision FLEX™ Kit K8008 (DAKO) or K5005 (Dako) according to standard protocols. Expression levels were assessed and scored by three experienced investigators (AzH, MAH, DR). The results of the HPyV7 LTag expression using the 2 t10 antibody have been described previously [6]. In the epithelial cells of 17 thymomas (46 %) marked LTag expression was found. The expression of LTag was in good agreement with earlier performed HPyV7-DNA PCR and/or the HPyV7-FISH [7].

### Statistics

Statistical analysis was performed with SPSS 20.0 statistical software (SPSS Inc., Chicago, IL, USA). Dichotomous variables are expressed as absolute numbers and percentages (%) and were compared using the chi-square test or Fisher’s exact test as appropriate. The Spearman’s rank correlation was used in non-parametric data to study the associations between different variables. Statistical significance was considered with the probability value of *P* < 0.05.

### Ethical approval

All specimens were collected at the Department of Pathology of the MaastrichtUniversity Medical Centre, for the Maastricht Pathology Tissue Collection, which includes ethical approval.All use of tissue and patient data was in agreement with the Dutch Code of Conduct for ObservationalResearch with Personal Data (2004) and Tissue (2001, www.fmwv.nl).

## Results

### Correlation between pRb and phosphorylated Rb expression in human thymomas and thymic carcinomas and HPyV7

**Fig. 1:**
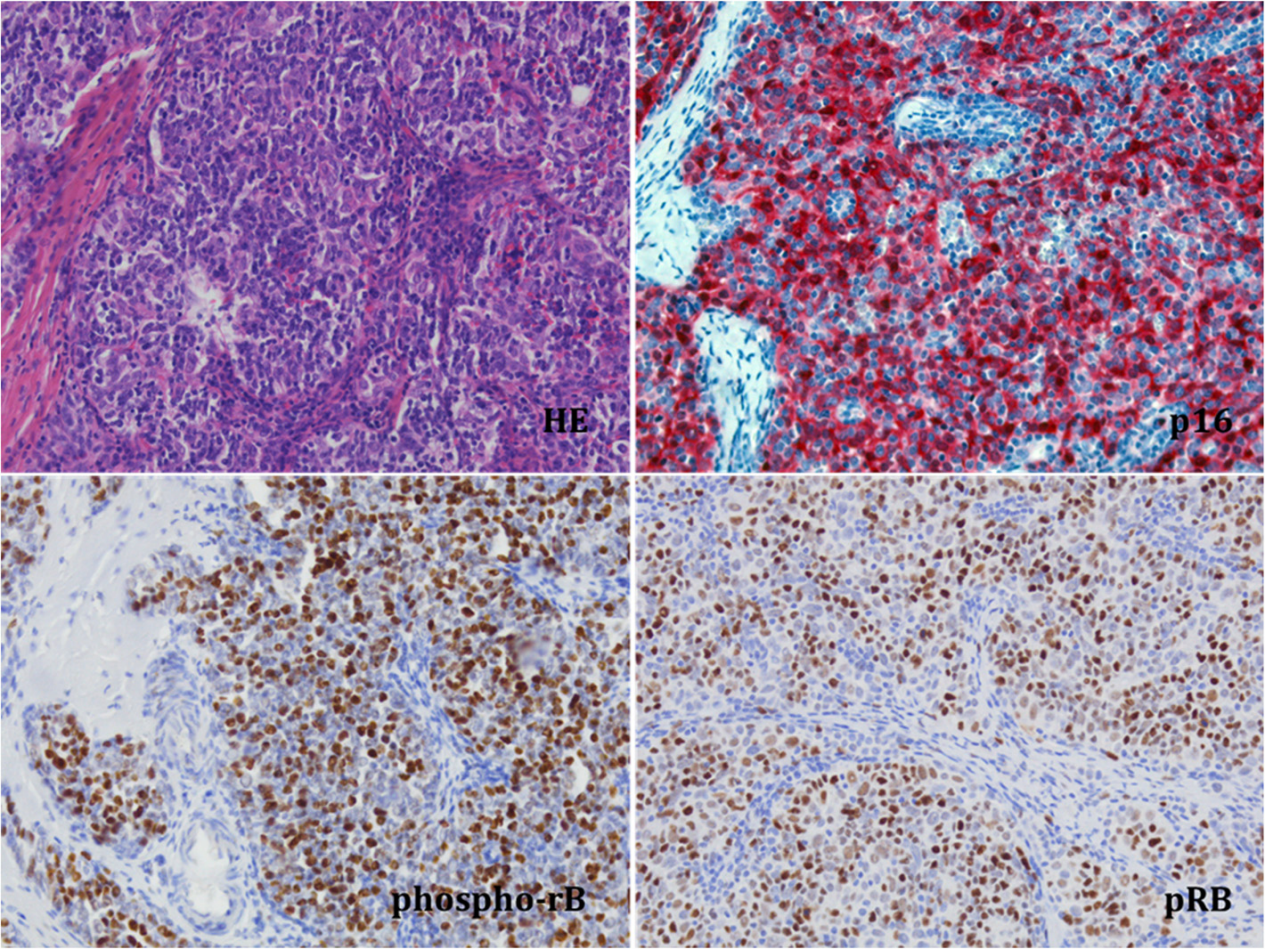
20X magnification images of thymoma 1–15 (Table 1). Upper left: Hematoxylin and eosin (H.E.) staining of a B3 thymoma revealing only few intraepithelial lymphocytes; upper right:immunohistochemical staining confirms strong (++) nuclear and cytoplasmatic expression of p16 (*red color*); lower left: expression of phospho-Rb is restricted to the nucleus of the thymic epithelial cells (*brown color*); lower right: expression of specific pRb is also restricted to the nucleus of the thymic epithelial cells (*brown*)

Thirty-five thymomas (94.6 %) showed specific expression of pRb within the neoplastic epithelial thymic cells (Fig. 1d). Phospho-Rb was observed in 31 thymomas (83.8 %) within the same cell compartment as pRb (Fig. 1c). No correlation between phospo-pRb expression and presence of HPyV7 could be established. In addition, no correlation was observed between the expression of pRb or phospho-Rb and WHO staging, Masaoka-Koga staging or the presence of MG.

### Correlation between HPyV7 and p16 expression in human thymomas and thymic carcinomas

Nuclear and cytoplasmic p16 expression [27] was detected in 19 thymomas (51.4 %) mainly within the neoplastic epithelial cells but also in dendritic cells as had been described [28]. Both thymic carcinomas did not reveal any p16 expression. No correlation between the immunoreactivity of p16 and WHO staging, Masaoka-Koga staging or the presence of MG was established. Of the 17 patients positive for large LTag, 9 patients (52.9 %) showed expression of p16. However, p16 was also detected in 10 patients (50 %) negative for LTag (Table 3). Three patients (8.1 %) showed very strong expression of p16 (++) (Fig. 1b). No differences regarding p16 expression levels were observed between LTag positive and negative thymomas. Therefore, the presence of HPyV7 was not associated with p16 expression.

**Table 3:** Absence of a correlation between P16 and HPyV7 in early and late stage thymoma and thymic carcinoma

HPyV7 IHC 2 t10				
	Masaoka– Koga I or II		Masaoka–Koga III or IV	
	–	+	–	+
P16				
–	6	8	5	1
+	10	7	0	2
Double positive		15/31		2/8
		48.4 %		25.0 %

### Follicular hyperplasia

PRb was detected in all 20 follicular hyperplasias in the lymphocytes. Phospho-Rb was also expressed in the lymphocytes in all follicular hyperplasias however, with a much lower intensity.

## Discussion

The presence of HPyV7 and the expression of the viral LTag were detected in the epithelial cells of human thymic epithelial tumors by PCR, FISH and IHC [6]. Yet, no information concerning the oncogenic capacity of HPyV7 is available. Of the 12 known human polyomaviruses only MCPyV has proven oncogenic capacity [11]. Expression of p16 has been detected in human thymic epithelial tumors on the transcriptional and translational level [21, 23, 24]. Since expression of p16 has been proposed as a potential surrogate marker for the presence and involvement of tumor viruses e.g., HPV, we investigated in as much p16 expression in human thymic epithelial tumors correlate with the presence of HPyV7 or expression of its LTag. However, we detected no correlation between the expression of LTag and p16 in human thymic epithelial tumors. Next, we investigated the co-expression of pRb/phospho-Rb and LTag because pRb is a major G1 checkpoint, which blocks S-phase entry and cell growth. However, no correlation between the immunoreactivty of pRB and/or phospho-Rb and LTag was observed.

Interestingly, we did not detect a specific pattern of p16 or pRB expression in relation to the invasiveness of thymomas. In our study 17/31 (54.8 %) of Masaoka-Koga Stage I or II and 2/8 (25 %) of Masaoka-Koga stage III or IV thymic epithelial tumors were double positive for P16 and pRb. This is in contrast with Hirabayashi et al. who reported that inactivation of p16 or RB could play a role in the progression of thymomas [23]. These differences are most likely a result of a distinct classification of non-invasive and invasive thymomas e.g., Masaoka stage II was counted as non-invasive in our study because of the little difference in overall survival between stage I and II [29]. More recently, Omatsu et al. showed that increasing malignancy is molecularly paralleled by a stepwise increase of p16 expression [24]. However, the difference within expression of p16 was only shown between thymic carcinomas and thymomas, without differences in expression level within thymomas. In our series only two thymic carcinomas were included and they were both negative for p16 expression. Interestingly, there were 3 patients (8.1 %) with strong immunoreactivity of p16 (++), none of these patients showed expression of LTag of HPyV7. As HPV infection has been ruled out as a possible cause for this p16 overexpression [21] these findings might suggest a role in virology.

Diverse human viruses including Poliovirus and oncogenic γ-herpesvirus Epstein Barr Virus have been detected in human thymic epithelial tumors [1, 2] on the search for the role of viruses in the pathogenesis of MG. Yet, these results need to be confirmed in a larger number of thymic epithelial tumors

## Conclusions

In conclusion, in this study we found no correlation between the presence of HPyV7 and pRb, phospho-Rb and p16 in human thymic epithelial tumors.

## Consent

All specimens were obtained from the Maastricht Pathology Tissue Collection. Alluse of tissue and patient data was in agreement with the Dutch Code of Conduct for ObservationalResearch with Personal Data (2004) and Tissue (2001, www.fmwv.nl), which includes informed patientconsent.

## Abbreviations

FISH: Fluorescence in situ hybridization
HPV: Human Papilloma Virus
HPyV7: Human Polyomavirus 7
IHC: Immunohistochemistry
LTag: Large T antigen
MCPyV: Merkelcel Polyoma virus
MG: Myasthenia gravis
PCR: Polymerase chain reaction
Phospho- Rb: Phosphoralized Retinoblastoma
pRb: Protein Retinoblastoma
STag: Small T antigen
WHO: World Health Organization
